# Phyllodes Tumor of the Breast: A Case Report Regarding the Importance of Fast Interdisciplinary Management

**DOI:** 10.3390/reports8010017

**Published:** 2025-02-02

**Authors:** Horia-Dan Lișcu, Andreea-Iuliana Ionescu, Iman Mologani, Nicolae Verga

**Affiliations:** 1 Department of Oncological Radiotherapy and Medical Imaging, “Carol Davila” University of Medicine and Pharmacy, 050474 Bucharest, Romania; horia-dan.liscu@drd.umfcd.ro (H.-D.L.); nicolae.verga@umfcd.ro (N.V.); 2 Radiotherapy Department, Colțea Clinical Hospital, 030167 Bucharest, Romania; iman.mologani@rez.umfcd.ro; 3 Department of Medical Oncology, Colțea Clinical Hospital, 030167 Bucharest, Romania

**Keywords:** malignant, phyllodes tumor, breast cancer, radiotherapy, case report

## Abstract

**Background and clinical significance:** Phyllodes tumors (PTs) are rare stromal neoplasms originating in the connective tissue of the breast, distinct from carcinomas that arise from the ducts or lobules. These tumors exhibit a broad spectrum of morphologic features and are traditionally classified as benign, borderline, or malignant. **Case presentation:** We present the case of a 71-year-old female diagnosed with a malignant PT and treated at our hospital. The patient noticed a gradually enlarging lump in her right breast over several months. Mammography was inconclusive, but an ultrasound later revealed a lobulated, firm mass, classified as BIRADS 5. Physical examination identified a 20 cm mass, and core needle biopsy suggested a borderline PT. Following lumpectomy, pathology confirmed a malignant tumor with narrow surgical margins (0.1 cm). Although mastectomy was recommended to achieve wider margins, the patient opted for adjuvant radiotherapy. She received 50 Gy in 25 fractions to the whole breast, followed by a 16 Gy boost to the tumor bed in 8 fractions. The treatment was well tolerated and completed successfully. Initially, the patient’s therapeutic management was delayed due to a combination of personal and organizational factors. However, the process was later streamlined through the use of a novel digital tool developed to facilitate the entire patient journey within our hospital system. **Conclusions:** This case highlights the diagnostic complexities of PTs, the critical need for effective collaboration between specialties, and the importance of timely treatment planning for optimal patient outcomes.

## 1. Introduction and Clinical Significance

Phyllodes tumors (PTs) of the breast are an uncommon type of fibroepithelial neoplasm, representing 0.3–1% of all breast tumors, with a reported incidence of 1 in 100,000 [1]. They most commonly occur in women of middle age, with an average age at diagnosis ranging from 40 to 50 years [2]. Their histological and radiographic features are similar to those of fibroadenomas, making it difficult to reach a definitive diagnosis through core needle biopsy. In many cases, the diagnosis of phyllodes may only be confirmed after surgical excision [3].

This case presentation addresses a common issue in countries where public healthcare systems are underfunded, leading to delays or interruptions in access to appropriate oncological treatment [4]. In this instance, we present a female patient with a PT who, for various reasons, experienced delays in completing her full oncological treatment. However, she was able to benefit from a new, developed in-house digital solution that mitigated earlier treatment planning delays. Not only the low incidence of this condition but also the treatment timeline, the tools that we used to facilitate the process and the therapeutic choice, taking the patient’s needs and choices into consideration, make this case special.

The treatment of PTs consists mainly of surgical resection. The current NCCN guideline bases treatment recommendations on tumor histology [5]. If the growth is classified as benign, an excisional biopsy is recommended. A wide excision accompanied by radiation therapy is the recommendation for borderline or malignant PTs [6]. The excisional biopsy entails the full resection of the mass, though it is not intended to secure clear surgical margins, whereas the wide resection is supposed to provide surgical margins larger than 1 cm. Narrow surgical margins may be associated with a higher risk of local recurrence, but if this target is not achieved after performing a partial mastectomy, conducting a second surgery is not mandatory. The role of adjuvant radiotherapy in the treatment of malignant PTs is intended to decrease local recurrence, providing an improved local control. No systemic chemotherapy agent is recommended to be used in non-metastatic PTs [5].

## 2. Case Presentation

We present the case of a 71-year-old female, with no relevant medical history, presenting with a malignant PT in her right breast, investigated and further treated in Colțea Clinical Hospital in Bucharest, Romania.

The patient noticed a gradually growing mass in her right breast for a few months. She decided to investigate the tumor in the radiology department of our hospital, receiving a mammography in May 2023. However, a precise assessment of the right breast was unsuccessful due to the size of the tumoral mass. An ultrasound examination of the tumor was recommended for the patient. She received the ultrasound in September 2023, which showed a lobulated mass, of hard consistency, associated with inflammatory lesions, especially in the lower quadrants of the right breast. The lesions were classified as BIRADS 5. She was further investigated in the outpatient surgery clinic of our hospital. Physical examination showed a voluminous tumoral mass measuring approximately 20 cm, located in the inferior quadrants of the breast. The overlying skin presented modified texture and color, prominent vascular aspect, and non-adherence to the underlying tissue. The surgeon performed a core needle biopsy. The histopathological and immunohistochemical examination results were available the following month, and they showed that the tumor was most likely a borderline PT.

In October 2023, the patient received a CT scan as part of the oncologic work-up, showing the breast tumor and a circumferential thickening of the pylorus. She was further advised to undergo an upper gastrointestinal endoscopy, which revealed esophagitis (Los Angeles grade A), antral gastritis and pre-pyloric ulcers (Forest III). Multiple biopsies were taken. This finding was incidental, with no impact on the oncological therapeutic decision, but it contributed to the delay in the treatment timeline.

In January 2024, the surgical team at our hospital performed a lumpectomy, removing the tumor, but this time the pathology report classified the tumor as a malignant periductal stromal mass. The surgical margins were narrow (0.1 cm). Between the months of January and May, the patient did not take any action regarding her condition.

In May 2024, she was evaluated by our hospital’s medical oncology team. The multidisciplinary team recommended immunohistochemical analysis of the excised tumor. The results came in July, confirming that the PT was indeed malignant. Follow-up ultrasonography was performed in August, showing the postoperative aspect of the breast, with no evidence of a tumoral mass. Due to the narrow surgical margin, she was advised to undergo a second surgery, a mastectomy on her right breast, which she refused.

To continue her treatment and to achieve better local control, avoiding the probability of the tumor reoccurring, she agreed to receive radiotherapy. She was prescribed by the radiation oncology team of our hospital a dose of 50 Gy in 25 fractions on the whole breast, followed by a boost of 16 Gy in 8 fractions on the tumoral bed. The tumoral bed was highlighted by the surgeon during surgery by placing metal clips.

**Figure 1:**
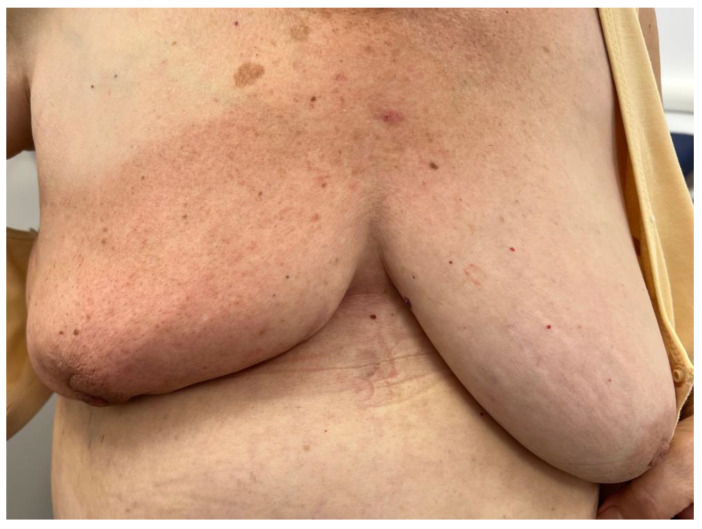
Picture taken after the last radiotherapy session showing grade I radiodermatitis of the right breast. The size of the breast should also be noted in comparison to the left one, considering the lumpectomy performed, which involved the removal of a large mass (>20 cm).

The 33 radiotherapy sessions were performed using conventional fractionation, 1 session per day from Monday to Friday, without treatment interruptions. The patient tolerated the radiotherapy without severe complications, developing only mild radiodermatitis (grade I, Figure 1) on the skin of her entire right breast and minor intertrigo underneath the breast. Under topical treatment with hyaluronic acid and steroid creams, the acute side effects of the radiotherapy were well controlled.

**Figure 2:**
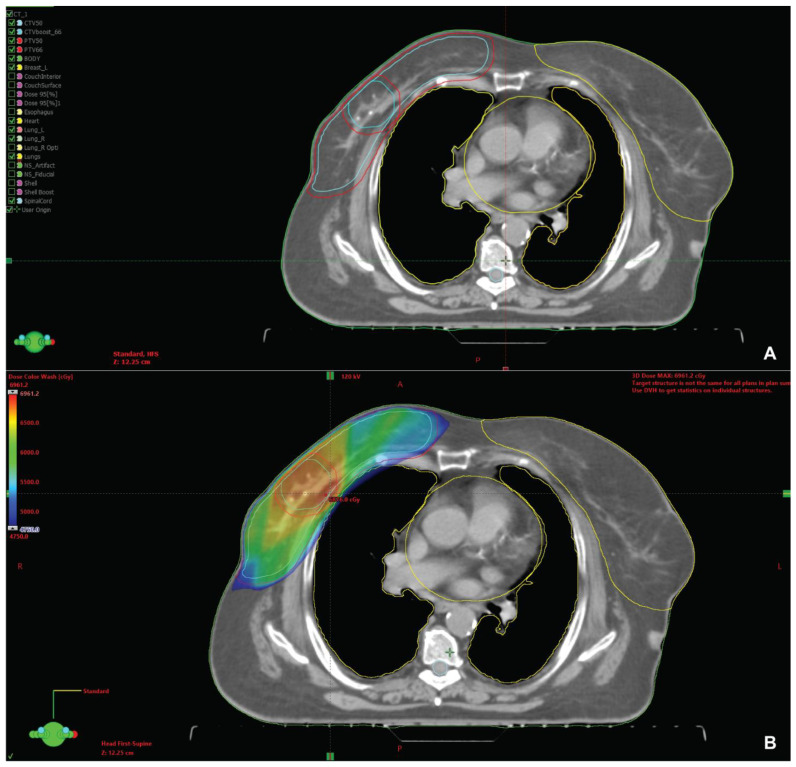
(**A**) Target volume delineation showing the CTV (light blue) and PTV (red) contouring for the whole breast and boost volumes. The organs at risk are contoured in yellow; and (**B**) color wash dose distribution in the PTV, in cGy. In the upper left corner of the image is represented a “color wash” scale of the distributed dose, starting with shades of red for the maximum doses administered (around 60 Gy), descending to shades of blue for low doses (around 50 Gy).

**Figure 3:**
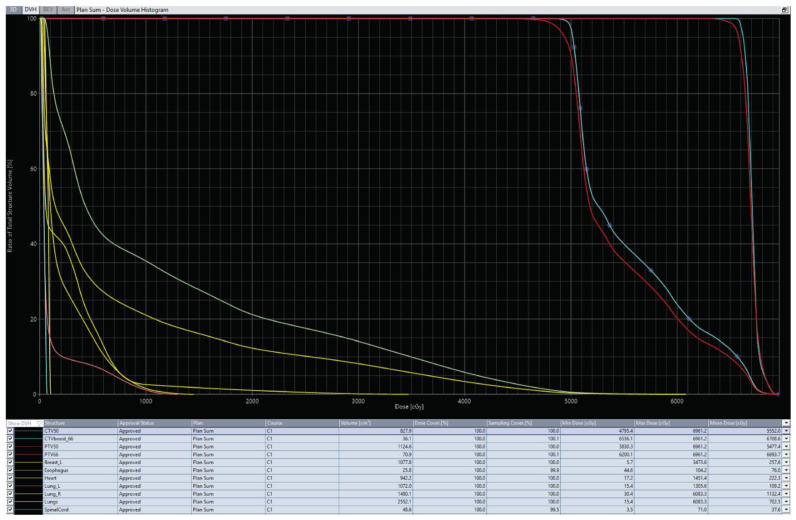
Dose–volume histogram (DVH) for the target volumes and organs at risk (OaR), in both graphical and tabular form. Red: CTV corresponding to volumes receiving 50 Gy and 66 Gy, respectively; light blue: PTV corresponding to volumes receiving 50 Gy and 66 Gy, respectively; shades of yellow: OaR doses (also illustrated in the table).

The irradiation technique we chose was VMAT using two arcs. Radiotherapy was delivered using a Varian Truebeam linear accelerator with 6 MV photon energy using an intensity-modulated radiation therapy (IMRT) technique. The treatment plan for the whole breast delineation followed the RADCOMP Breast Atlas protocol and 2 cm isotropic expansion around the central point defined by the metal clips covering the boost volume (Figure 2). The dose–volume histogram is presented in Figure 3.

With the financial support of funding awarded by the Romanian Ministry of Research, Innovation and Digitalization, the authors of this article have developed a digital application aiming to help the physicians to schedule interdisciplinary in-hospital consultations. Although still in the testing phase, this application was utilized for this patient, enabling quick and efficient scheduling of interdisciplinary consultations.

**Figure 4:**
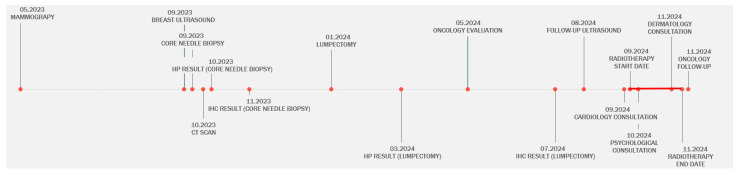
The timeline of the treatment planning for our patient, highlighting the disruptions to the scheduling of the medical care steps our patient experienced at the beginning.

The patient underwent a cardiology check-up 5 days before beginning radiation therapy to assess the cardiac function prior to treatment, a medical psychology consultation (routine for oncology patients), a dermatology consultation after her 25th radiotherapy session for the submammary intertrigo, and a medical oncology consultation for registering and stage evaluation a few days after the radiation therapy was completed. All these consultations were carried out in record time, just 1 day after scheduling through the app. This digital solution allowed all the remaining investigations to be carried out much faster, especially in the case of a patient who had already lost several critical months of disease progression (Figure 4).

The digital solution can be used on any type of computer or smartphone and allows hospital physicians, regardless of specialty, to assist the patient in scheduling interdisciplinary consultations through a “book a free slot” system. Thus, when the patient completes a consultation, the next step in the therapeutic management of the disease is already scheduled, avoiding the occurrence of prolonged pauses due to administrative or organizational problems. The advantage of having all the history available in digital format in this application allows the multidisciplinary team to consider all the clinical and paraclinical data observed independently by different physicians. Thus, the therapeutic decisions can be personalized, considering all the patient’s data but also the limited resources available in some hospitals.

## 3. Discussion

Phyllodes tumors are stromal tumors, unlike breast carcinomas, starting in the connective tissue of the breast, outside the ducts and lobules. They are known for their capacity to appear in a wide spectrum of morphology, with their biologic behavior varying from benign to malignant [7]. The World Health Organization (WHO) classifies PTs into benign, borderline, and malignant, comparing their characteristics to those of the fibroadenoma (Table 1). The criteria considered for this classification are the histologic characteristics, including the stromal cellularity and atypia, mitotic activity, stromal overgrowth, the appearance of the tumoral margins, and the presence of the malignant heterologous elements. The description of these subtypes also includes their distribution relative to all breast tumors and their relative proportion among all PTs [8].

### 3.1. Challenges in Diagnosing a Rare Disease

**Figure 5:**
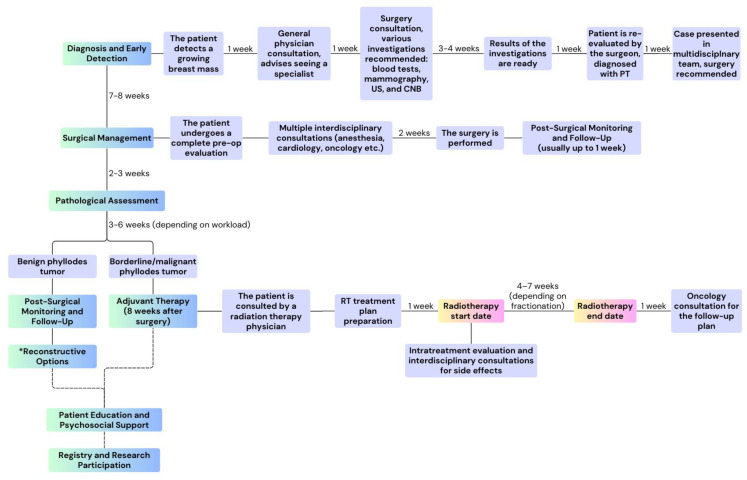
The ideal course of treatment for a borderline/malignant phyllodes tumor patient at our hospital, lasting for approximately 5–6 months. Abbreviation: US, ultrasound; CNB, core needle biopsy; PT, phyllodes tumor; RT, radiation therapy. *Reconstructive surgery is optional, it is not part of the treatment plan recommendations, but it might be taken into consideration by the patient for cosmetic reasons.

Managing rare diseases, like PTs, is far more complex than treating more common types of breast cancer. Figure 5 outlines the key steps the multidisciplinary team must consider when treating patients diagnosed with such conditions, along with the ideal timeline of a patient’s journey, from diagnosis to complete treatment, at our hospital. In this case, the process was extended due to personal reasons on the part of the patient and delays in securing appointments. However, the implementation of the application we developed helped streamline the process, and we believe that using this tool can further enhance even the ideal timeline.

Diagnosis and early detection of the disease can be challenging due to several factors. The histopathological similarities between benign PTs and fibroadenomas, as highlighted in Table 1, often complicate the correct identification of the tumor type and the assessment of the urgency of treatment. Another limitation noted in the literature is the misdiagnosis of the tumor grade when evaluating core needle biopsies [9], a challenge we also faced. Additionally, the relatively rare occurrence of PTs among all breast tumors increases the likelihood of an incorrect diagnosis being made.

**Figure 6:**
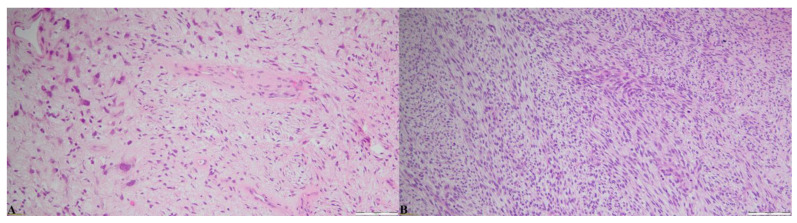
(**A**) Hematoxylin and eosin staining (20× magnification) of the core needle biopsy, showing moderate stromal cellularity and marked stromal atypia. (**B**) Hematoxylin and eosin staining (20× magnification) of the resected specimen, showing stromal overgrowth, marked to moderate stromal cellularity, marked stromal atypia and mitosis, and infiltrative tumor margins.

One intriguing finding in our case is that the core needle biopsy showed the borderline characteristics of the tumor, while the histopathological and immunochemistry examinations performed on the specimen obtained during the lumpectomy proved that the tumor was in fact malignant (Figure 6). This finding highlights the importance of assessing the whole tumoral mass and proving the limitations of a core needle biopsy. Most likely, the primary cause of this discrepancy between core needle biopsy and excision is the heterogeneity of the stromal component in PTs [9]. One study, conducted by Choi and Koo in 2011, compared the histopathological features of core needle biopsy and surgical excision specimens in 129 patients with surgically confirmed PTd. Five malignant PT cases were misdiagnosed via core needle biopsy. Two were identified as benign, and three were classified as borderline. Stromal cellularity and mitosis were notable parameters that exhibited greater discrepancies during grading [9].

The tumor should also be investigated through imaging modalities, such as ultrasound, mammography or MRI, for additional data regarding the diagnosis and for pre-surgical evaluation. The number of investigations requiring high-performance devices is considerable, and the process of a complete evaluation is especially hampered if the medical facility does not provide in-house all the forementioned examinations. Our hospital provides all the imaging modalities, but the large size of the tumor made it difficult for mammography to be performed. Therefore, an ultrasound was recommended, delaying the process.

### 3.2. Surgical Management

Surgical management should be planned only after a thorough evaluation and definitive diagnosis. This planning involves determining the optimal surgical margins to ensure local control and deciding between breast-conserving surgery and mastectomy, based on the tumor’s histopathological assessment [5]. The post-surgical monitoring and follow-up are essential to detect any possible recurrence. The risk stratification is based on the tumor grade and tailored follow-up plans should be made by the multidisciplinary team. The team should also discuss reconstructive options, especially if the patient wishes to improve the cosmetic outcomes of the surgery. In our case, adequate surgical margins were not attained; therefore, an additional surgery aiming to secure optimal borders was recommended. Since the patient refused to have another operation, adjuvant radiation therapy was the best treatment plan option.

### 3.3. Radiation Treatment

Adjuvant therapy should be performed if the PT is borderline or malignant. Radiation therapy proved its role in local control. The multidisciplinary team should also address strategies for managing potential recurrence. The rarity of metastatic cases further complicates the decision-making process for an appropriate treatment plan [10].

The literature on PTs is heterogeneous. Developing consensus guidelines based on the literature will support the provision of evidence-based care [11]. While benign PTs are known to respond excellently to surgical treatment, with high local control rates, malignant and borderline ones have higher chances of local recurrence or may metastasize. For patients with borderline PTs, radiotherapy is beneficial if the surgical margins are close or positive, even after optimal resection. Adjuvant radiotherapy shows a trend toward improved local control in malignant cases. However, a metastatic malignant PT typically has a poor prognosis [12].

The radiotherapy outcomes are also different depending on the type of PT. It has been proved that adjuvant radiation therapy does not bring any advantages in terms of the recurrence-free or overall survival of patients presenting with benign PTs, nor in patients with borderline PTs or malignant PT patients who received mastectomies [13]. In 2021, Boutrus et al. published a study where they evaluated 108 patients with all three types of PT from their clinic. Out of the total, 32 patients (30%) received adjuvant radiation therapy, comprising 3 from the benign group and 29 from the borderline/malignant group. Patients who had breast-conserving surgery with clear margins showed a significantly higher 5-year local recurrence-free survival (LRFS) when treated with adjuvant radiation compared to those who did not receive radiation (100% vs. 34.3%, *p* = 0.022). In contrast, among those who underwent mastectomy with clear margins (*n* = 18), the benefit in terms of the 5-year LRFS with adjuvant radiation therapy was not statistically significant (100% vs. 83%, *p* = 0.24) [13].

In past years, the importance of radiotherapy in treating PT of the breast was studied, along with multiple dose fractionation regimens. Adjuvant radiation has been shown to reduce local recurrence and may be recommended when recurrence could lead to significant morbidity. Margin-negative resection combined with adjuvant radiotherapy is highly effective in achieving local control of borderline and malignant PTs. The rate of local recurrence with adjuvant radiotherapy is significantly lower compared to that observed in patients who undergo margin-negative resection alone [6].

The standard radiation treatment for breast cancer typically uses conventional fractionation (1.8–2.0 Gy per fraction). Moderate hypofractionation (2.7 Gy per fraction) was not proved to be in any way superior to the conventional one [14,15,16]. Nonetheless, the literature contains a few case reports of PTs successfully treated with hypofractionated radiotherapy [17]. Considering the scarcity of mature data about the use of hypofractionation in PTs, this patient’s radiation oncologist chose to use conventional doses.

The biologically effective dose (BED) is calculated to compare different regimens, with conventional schedules yielding a BED of 73.1–75.0 Gy, while the hypofractionation regimen has a BED of 67.8 Gy. When planning the treatment of this patient, we opted for the standard treatment regimen, prescribing 50 Gy in 25 fractions on the whole breast, followed by a boost of 16 Gy in 8 fractions on the tumoral bed.

In 2023, Alvarez et al. conducted a retrospective review of 14 cases of PT. The patients were treated between 2015 and 2023. The authors described the outcomes of using postoperative radiotherapy with moderate dose escalation for patients with high-risk features. This included cases with borderline or malignant histologies, as well as benign tumors with narrow or positive surgical margins following surgery [18]. The patients received whole breast/chest wall irradiation, aiming for a BED above 90 Gy (between 90 and 102.6 Gy), slightly higher than the standard postoperative dose for mammary carcinoma but closer to that used for soft tissue sarcomas [19,20]. The target volumes did not include the regional lymph nodes. Four different radiotherapy regimens were used, with doses ranging between 2.0 and 3.6 Gy being administered in 15 to 33 fractions. The chosen radiation method was the three-dimensional (3D) one, except in one patient who received volumetric-modulated arc therapy (VMAT). The outcomes suggest that a moderate dose escalation is implementable, being a choice worth considering over the traditional one [18].

Due to the malignant characteristics of the PT identified in this patient, we were particularly attentive to any signs indicating metastasis in the body. Metastasis occurs in up to 22% of patients with a malignant PT at the time of presentation. The most frequent sites of metastasis include the lungs, followed by the bones, heart, and liver [21]. The updated guidelines recommend re-excision with wide margins to be performed if the PT reoccurs [5]. If we identify metastasis, the disease shall be treated following the guidelines for soft tissue sarcoma [5]. This approach should be a combination of the surgical excision of the metastasis, radiation therapy and systemic treatment, using an anthracycline-based regimen as a first-line treatment [22]. Unlike the treatment of invasive breast carcinoma, breast sarcomas do not benefit from CDK 4/6 inhibitors, either adjuvant or metastatic [23]. In 2023, Samii et al. published a systematic review of metastatic or recurrent malignant PT of the breast from 2010 to 2021. The review included 66 patients across 63 studies. Among them, 78.8% had distant metastatic disease, and 31.8% had locoregional recurrence. Surgical excision was the primary treatment, with some cases receiving radiotherapy or chemotherapy. The median survival was 24 months for distant metastases and 72 months for locoregional recurrence. Another author evaluated the life expectancy for patients with metastatic-stage PT, reaching an OS of less than 1 year [24] under conditions of a major risk of progression to organ failure and a low quality of life [25,26]. The treatment strategies for recurrent or metastatic malignant PT remain uncertain, and further research is required to identify effective approaches [10].

### 3.4. Patient Education, Research-Based Data and Multidisciplinary Collaboration

Patient education and psychosocial support are necessary given the patient anxiety following such a diagnosis. The rare nature of the disease and the treatment outcomes should be discussed with the patient, whose options and personal beliefs should always be taken into consideration when deciding on the treatment plan. The scarcity of PT makes it important to contribute to rare tumor registries for data collection and to encourage fellow physicians and patients to enroll in clinical trials to improve evidence-based treatment [10]. One important problem that we noticed was the lack of trials focusing on adjuvant radiotherapy treatment for PT. While there are several studies on adjuvant radiation therapy for breast cancer, those studies do not include PT. Due to the limited availability of randomized controlled trials or prospective studies caused by the relatively low incidence of PT, synthesizing real-world data can provide a sufficient body of evidence to help clinicians make informed decisions about the optimal treatment [27]. This topic warrants further research, and additional data, including fractionation regimens and treatment recommendations for PT metastasis, should be made available to physicians to enable more effective treatment planning.

The digital solution presented in this case offers a sophisticated approach to managing interdisciplinary consultations within hospital settings. The application, designed specifically for medical staff, replaces traditional communication methods, such as phone calls or handwritten book entries, with a seamless digital system. The user-friendly interface enables doctors to quickly schedule interdisciplinary consultations by selecting the specialty and the referring doctor, and inputting the patient’s relevant information. Upon submission, the requested specialist is notified, and they can either accept or reject the request, providing an efficient, organized way to coordinate patient care. This web application, built using Flask (Python) for backend processing, HTML and CSS for frontend rendering, and SQLAlchemy for database management, ensures that the entire process runs smoothly, from appointment scheduling to the notifications received by the specialist. The design’s intuitive nature, coupled with the client–server architecture, guarantees that medical professionals can easily navigate the system, minimizing the time spent on administrative tasks.

The application was successfully employed for the patient in this case study, allowing for the swift scheduling of necessary consultations in cardiology, medical psychology, dermatology, and oncology, all within one day of initiating the request. This rapid response is a significant advantage, particularly in the context of underfunded healthcare systems where administrative delays often hinder timely access to specialist care. Prior to the implementation of the digital solution, coordinating these consultations would have required multiple phone calls and manual scheduling, which typically results in longer waiting times, risking missed or delayed appointments. The efficiency introduced by this web application not only eases the coordination of the care path but also ensures that critical medical evaluations and urgent treatments proceed without unnecessary interruptions, which is crucial for patients who have already experienced delays in receiving medical care. This digital solution was only used in the second part of the treatment (before, during and after radiotherapy) and saved an estimated one week of the patient’s time just during radiotherapy. If it had been used from the very beginning of the therapeutic management, at which time the application was still under development, certainly all the therapeutic procedures before radiotherapy would have been performed more efficiently. If classical scheduling methods would have been used, the patient would have been scheduled 1–4 days apart for each interdisciplinary consultation during radiotherapy, causing delays. By using the “book a free slot” function, it was even possible to perform the consultations on the same day.

While the clinical benefits of this digital solution are evident, it is important to consider the privacy and security of the sensitive medical data handled by the application. As the healthcare sector increasingly embraces digital tools, maintaining robust data protection measures is essential. The application adheres to stringent privacy regulations, ensuring that patient data are securely stored and transmitted. The use of encrypted communication between the client (user interface) and the server guarantees that patient information remains confidential, in line with established data protection laws. This focus on security ensures that the app not only enhances operational efficiency but also safeguards the integrity and privacy of patient data—an essential consideration in today’s increasingly digital healthcare landscape.

Collaboration between surgeons, oncologists, radiation therapy doctors and pathologists for optimal management is crucial. Therefore, we consider that the application we developed is a useful tool to speed up the process of diagnosing and treating any disease, but more importantly, the rare ones.

## 4. Conclusions

The diagnosis of malignant phyllodes tumors is rare, necessitating a multidisciplinary approach to ensure comprehensive investigation and appropriate treatment. Equally important is the inclusion of the patient’s preferences in the decision-making process. In the case we treated, the patient declined a second surgery to achieve wider surgical margins and instead opted for adjuvant radiotherapy. Numerous studies have demonstrated the efficacy of adjuvant radiation in enhancing local control and improving disease-free survival. The treatment in this case was successfully completed without complications. A significant factor contributing to the positive outcome was the use of a newly developed in-house digital tool, which streamlined access to additional consultations, facilitating a more efficient and coordinated treatment plan.

## Figures and Tables

**Table 1:** Histological features of benign, borderline, and malignant phyllodes tumors compared to the features of the fibroadenoma (latest WHO classification, 2019).

Histological Feature	Fibroadenoma		Phyllodes Tumors	
		Benign	Borderline	Malignant
Tumor border	Well defined	Well defined	Well defined, may be focally permeative	Permeative
Stromal cellularity	Variable, scant to uncommonly cellular, usually uniform	Cellular, usually mild, may be non-uniform or diffuse	Cellular, usually moderate, may be non-uniform or diffuse	Cellular, usually marked, and diffuse
Stromal atypia	None	Mild or none	Mild or moderate	Marked
Mitotic activity	Usually none, rarely low	Usually low: <2.5 mitoses/mm^2^ (<5 per 10 HPFs)	Usually frequent: 2.5 to <5 mitoses/mm^2^ (5–9 per 10 HPFs)	Usually abundant: ≥5 mitoses/mm^2^ (≥10 per 10 HPFs)
Stromal overgrowth	Absent	Absent	Absent (or very focal)	Often present
Malignant heterologous elements	Absent	Absent	Absent	May be present
Distribution relative to all breast tumors	Common	Uncommon	Rare	Rare
Relative proportion of all phyllodes tumors	n/a	60–75%	15–26%	8–20%

Abbreviation: HPF, high-power field; n/a, not applicable.

## Data Availability

The data from this study are available upon request from the authors. Due to ethical considerations, the data are not publicly available.
